# The Cardiovascular Risk Paradox in Normal-Weight Steatotic Liver Disease Beyond Body Mass Index–Based Detection

**DOI:** 10.1016/j.gastha.2026.100988

**Published:** 2026-05-05

**Authors:** Masashi Hirooka, Teruki Miyake, Ryo Yano, Yoshiko Nakamura, Yuki Okazaki, Toyoki Shimamoto, Atsushi Yukimoto, Yasunori Yamamoto, Takao Watanabe, Osamu Yoshida, Yoshio Tokumoto, Masanori Abe, Takeru Iwata, Yoichi Hiasa

**Affiliations:** 1 Total Medical Support Center, Ehime University Hospital, Toon, Ehime, Japan; 2 Department of Gastroenterology and Metabology, Ehime University Graduate School of Medicine, Toon, Ehime, Japan; 3 Ehime General Health Care Association, Matsuyama, Ehime, Japan

**Keywords:** Metabolic Dysfunction, Cardiovascular Disease, Hepatic Steatosis, Precision Medicine, Risk Stratification

## Abstract

**Background and Aims:**

Cardiovascular screening largely relies on anthropometric metrics and overlooks normal-weight individuals with metabolic dysfunction, particularly those with steatotic liver disease (SLD). This gap may leave a substantially high-risk population undetected. Therefore, this study assessed the prevalence of cardiovascular disease (CVD) across 4 distinct metabolic phenotypes defined by body mass index (BMI) and hepatic steatosis status.

**Methods:**

We analyzed 34,848 Japanese adults enrolled in a community health screening program (2007–2010). Participants were classified into 4 metabolic phenotypes based on BMI and steatosis using the fatty liver index. The primary outcome was prevalent CVD. Logistic regression and machine learning models were used to compare the cardiovascular risk across phenotypes.

**Results:**

Normal-weight individuals with SLD (NWSLD; n = 496, 1.4%) demonstrated the highest CVD prevalence (3.63%) vs healthy controls (0.72%) and obese individuals with SLD (1.76%). After multivariable adjustment, NWSLD remained strongly associated with CVD (odds ratio: 3.63; 95% confidence interval: 2.11–5.88; *P* < .001). Incorporating NWSLD improved prediction over BMI-based models (C-statistic 0.790 vs 0.781; *P* < .001) and showed favorable clinical efficiency (number needed to treat = 34).

**Conclusion:**

NWSLD represents an unrecognized yet highly vulnerable cardiovascular phenotype, challenging BMI-centric risk assessment and supporting metabolic-based precision screening.

## Graphical abstract

**Figure:**
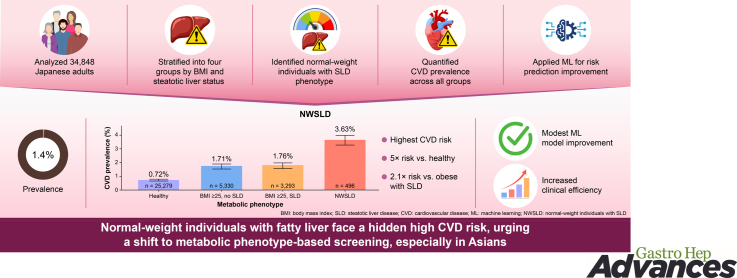

## Introduction

Cardiovascular risk assessments remain largely body mass index (BMI)-centric; however, these models often fail to accurately capture metabolic dysfunction. Despite increasing awareness of BMI-independent metabolic risk, current models still miss millions of individuals with a low BMI but high metabolic burden.^1^ Many normal-BMI individuals exhibit metabolic abnormalities, leading to frequent misclassification by conventional risk models.^2^

The concept of normal-weight individuals with metabolic dysfunction emerged in the 1980s.^3^ These individuals demonstrate visceral adiposity, insulin resistance, atherogenic dyslipidemia, and chronic low-grade inflammation, despite appearing healthy by conventional anthropometric criteria. However, population-scale validation of cardiovascular risk in such individuals remains limited, with most studies confined to small cohorts or focusing on metabolic rather than cardiovascular endpoints.^4^

Steatotic liver disease affects 25%–30% of adults globally, representing a key marker of metabolic dysfunction that transcends BMI boundaries.^5^ Hepatic steatosis strongly predicts cardiovascular events independently of traditional risk factors^6^; however, its interaction with BMI in determining cardiovascular risk remains incompletely understood. Asian populations may be particularly susceptible to metabolic dysfunction at lower BMI thresholds,^7^ highlighting the relevance of studying normal-weight individuals with steatotic liver disease (NWSLD).

Current cardiovascular risk calculators rely predominantly on BMI-based classifications, potentially overlooking high-risk individuals who appear metabolically healthy by anthropometric measures.^8^ This knowledge gap has immediate clinical implications, as early identification of high-risk individuals enables targeted cardiovascular prevention.^9^ The prevalence and cardiovascular risk profile of NWSLD disease in large real-world health screening populations remain unknown.

We hypothesized that individuals with NWSLD would exhibit disproportionately elevated cardiovascular risk compared to individuals with elevated BMI, challenging traditional BMI-centric models. Therefore, we examined the prevalence of cardiovascular disease (CVD) across 4 distinct metabolic phenotypes defined by BMI and hepatic steatosis status.

## Methods

### Study Population and Design

This cross-sectional study utilized data from a large-scale health screening program conducted in Japan between 2007 and 2010. The program included voluntary participants aged ≥20 years who underwent comprehensive annual health examinations, representing a diverse, real-world population. From an initial cohort of 75,781 participants, systematic exclusions were applied to ensure data completeness and analytical validity. We excluded 10,599 participants with missing BMI or fatty liver index (FLI) data, and 30,334 participants with incomplete covariate information, yielding a final analytic sample of 34,848 participants. To assess potential selection bias arising from missing data, we compared baseline characteristics between participants included in the complete-case analysis and those excluded due to missingness. Standardized mean differences were calculated to quantify the magnitude of between-group differences. Because certain metabolic characteristics differed between included and excluded participants (Supplementary Table 1), we conducted a prespecified sensitivity analysis using multiple imputation by chained equations to evaluate the robustness of the primary findings. The study population included participants spanning diverse demographic, socioeconomic, and health status categories. All participants underwent standardized medical examinations, including anthropometric measurements, laboratory assessments, and clinical evaluations according to established health screening protocols.

This study was approved by the institutional review board of Ehime University Graduate School of Medicine (approval number: 1104005). All participants provided written informed consent to participate in the health screening program and to the use of their deidentified data for research purposes. The study was conducted in accordance with the Declaration of Helsinki (2024) and Good Clinical Practice guidelines.

### Metabolic Phenotype Classification

Participants were classified into 4 mutually exclusive metabolic phenotypes based on BMI and hepatic steatosis status.

1. Healthy (BMI <25 kg/m^2^)
2. Elevated BMI (≥25 kg/m^2^) without steatotic liver disease
3. Elevated BMI (≥25 kg/m^2^) with steatotic liver disease
4. Normal-weight individuals (BMI <25 kg/m^2^) with steatotic liver disease (NWSLD)

We used an Asian-specific BMI threshold of 25 kg/m^2^ to define overweight, consistent with the World Health Organization (WHO) recommendations.

### Waist Circumference Measurement and Classification

Waist circumference (WC) was measured at the umbilical level in the standing position using a nonelastic tape. According to the WHO Asia-Pacific thresholds, a high WC was ≥90 cm in men and ≥80 cm in women. We further stratified individuals with NWSLD into high WC and normal WC subgroups to explore the contribution of central obesity to CVD prevalence.

### Liver Fibrosis Estimation

The fibrosis risk was estimated using the Fibrosis-4 (FIB-4) index, calculated as (age × aspartate aminotransferase)/(platelet × √alanine aminotransferase). Participants were categorized as FIB-4 <2.67 (no advanced fibrosis) or ≥2.67 (advanced fibrosis). The distribution across metabolic phenotypes is shown in Table 1.

**Table 1:** Baseline Characteristics by Metabolic Phenotype

Characteristic	Healthy (n = 25,729)	Elevated BMI, no SLD (n = 5330)	Elevated BMI + SLD (n = 3293)	NWSLD (n = 496)	*P* value
Age (y)	45.9 ± 8.8	48.2 ± 8.4	47.8 ± 8.1	48.9 ± 8.2	*<*.*001*
Male sex, n (%)	12,144 (47.2)	2814 (52.8)	2034 (61.8)	360 (72.6)	*<*.*001*
BMI (kg/m^2^)	21.8 ± 2.1	27.2 ± 2.8	28.1 ± 3.2	22.9 ± 1.8	*<*.*001*
Waist circumference (cm)	74.2 ± 7.8	87.3 ± 8.2	91.4 ± 9.1	81.2 ± 6.9	*<*.*001*
FLI	15.2 ± 12.8	22.8 ± 15.4	85.7 ± 23.1	78.4 ± 18.2	*<*.*001*
Advanced fibrosis (FIB-4 ≥2.67)	31	46	29	0	*<*.*001*
Total cholesterol (mg/dL)	195 ± 32	201 ± 34	208 ± 36	203 ± 35	*<*.*001*
Triglycerides (mg/dL)	89 ± 54	125 ± 78	168 ± 102	143 ± 89	*<*.*001*
HDL cholesterol (mg/dL)	65 ± 16	58 ± 14	52 ± 12	55 ± 13	*<*.*001*
LDL cholesterol (mg/dL)	115 ± 28	122 ± 31	129 ± 33	123 ± 30	*<*.*001*
HbA1c (%)	5.1 ± 0.4	5.3 ± 0.6	5.6 ± 0.8	5.4 ± 0.7	*<*.*001*
ALT (U/L)	16 ± 12	22 ± 16	35 ± 24	29 ± 19	*<*.*001*
AST (U/L)	20 ± 8	23 ± 10	28 ± 15	25 ± 11	*<*.*001*
GGT (U/L)	21 ± 18	31 ± 28	48 ± 42	42 ± 35	*<*.*001*
CVD, n (%)	184 (0.72)	91 (1.71)	58 (1.76)	18 (3.63)	*<*.*001*
Diabetes, n (%)	2161 (8.4)	863 (16.2)	942 (28.6)	100 (20.2)	*<*.*001*
Hypertension, n (%)	4580 (17.8)	1887 (35.4)	1722 (52.3)	211 (42.5)	*<*.*001*

FIB-4 = (age × AST)/(platelet × √ALT); advanced fibrosis defined as ≥2.67. *P* value <.05 was considered statistically significant and formatted in italics.

ALT, alanine aminotransferase; AST, aspartate aminotransferase; GGT, gamma-glutamyltransferase; HbA1c, hemoglobin A1c; HDL, high-density lipoprotein; LDL, low-density lipoprotein.

### Hepatic Steatosis Assessment

Hepatic steatosis was defined using the FLI, a validated algorithm that incorporates BMI, WC, triglycerides, and gamma-glutamyltransferase levels.^10^ The FLI ranges from 0 to 100, with ≥60 indicating hepatic steatosis.^11^^,^^12^ This threshold has been extensively validated in Asian populations and is strongly correlated with liver biopsy findings. While magnetic resonance imaging proton density fat fraction offers precise quantification,^13^ the FLI remains the most practical tool for large-scale epidemiological research. While FLI includes BMI and WC as components, it also integrates biochemical markers of metabolic dysfunction (triglycerides and gamma-glutamyltransferase), thereby capturing hepatic and metabolic characteristics beyond mere anthropometric measures. In this study, NWSLD was defined by the presence of steatosis within BMI strata rather than by BMI itself, minimizing concerns regarding direct circularity between exposure definition and stratification.

### Outcomes

The primary outcome was the prevalence of CVD, characterized by a history of coronary artery disease or cerebrovascular disease.

Coronary artery disease was defined as cases in which diagnosis and treatment were confirmed by coronary angiography and/or coronary revascularization procedures. These events were primarily identified via formal notification from the treating medical institution to the health screening center. In instances where institutional notification was unavailable, cases were identified based on participant self-report of physician-diagnosed coronary artery disease.

Cerebrovascular disease was defined as cases confirmed by imaging-based diagnosis and treatment. Similar to coronary disease, these cases were primarily identified through notification from the treating institution to the health screening center, with participant self-report used when institutional notification was not available.

Peripheral arterial disease was not included in the composite CVD outcome.

Secondary outcomes included diabetes mellitus (fasting glucose ≥126 mg/dL, hemoglobin A1c ≥6.5%, or antidiabetic medication use) and hypertension (systolic blood pressure ≥140 mmHg, diastolic blood pressure ≥90 mmHg, or antihypertensive medication use).

The specific questionnaire items used for CVD classification are listed in Supplementary Table 2.

### Statistical Analyses

Baseline characteristics were compared across metabolic phenotypes using analysis of variance for continuous variables and chi-square tests for categorical variables. Continuous variables are presented as mean ± standard deviation, and categorical variables as numbers (percentages).

Logistic regression models were used to evaluate cardiovascular risk across metabolic phenotypes, progressively adjusting for age and sex (model 1); cardiometabolic risk factors, including diabetes and hypertension (model 2); and comprehensive behavioral and laboratory covariates, including smoking status, alcohol consumption, exercise habits, and laboratory parameters (model 3).

We developed and compared 4 predictive models using traditional statistical approaches and machine learning (ML) techniques: (1) conventional BMI-based model, (2) steatotic liver disease-enhanced model, (3) NWSLD phenotype model, and (4) comprehensive model with interaction terms. ML approaches included random forest algorithms with 10-fold cross-validation to optimize hyperparameters and assess model generalizability. This ML subanalysis was exploratory and hypothesis-generating, intending to evaluate whether adding hepatic phenotype information (eg, NWSLD) improves cross-sectional discrimination for prevalent CVD beyond BMI-based models; it was not intended for clinical implementation.

Model performance was evaluated using C-statistics with 95% confidence intervals (CIs), likelihood ratio tests, and net reclassification improvement. Calibration was assessed using Hosmer–Lemeshow tests and calibration plots across risk deciles. Clinical impact was quantified using the number needed to treat (NNT), population-attributable risk, and absolute risk differences, with NNT calculated as 1/(risk difference) compared with the healthy reference group. Because this study is cross-sectional and evaluates prevalent CVD, NNT was derived from absolute differences in CVD prevalence and should be interpreted as a cross-sectional clinical efficiency metric (ie, number needed to identify one additional prevalent case), rather than a causal estimate of future event prevention.

Sensitivity analyses examined robustness across different FLI thresholds (50, 60, and 70) and BMI cutoff points (23, 24, and 25 kg/m^2^). Multiple imputation using chained equations was performed as a sensitivity analysis to address potential selection bias due to missing data, with estimates pooled according to Rubin’s rules. Additionally, we repeated the logistic regression after excluding participants with a prior history of CVD to evaluate potential reverse causation. To assess alcohol-related confounding, we excluded participants with high alcohol intake (≥30 g/d for men and ≥20 g/d for women) according to the Japanese Society of Hepatology criteria and repeated the analyses.

All analyses were performed using R version 4.3.0 (R Foundation for Statistical Computing). CIs (95%) were calculated using bootstrap resampling (n = 1000). Statistical significance was set at *P* < .05.

## Results

### Study Population and Phenotype Distribution

**Figure 1:**
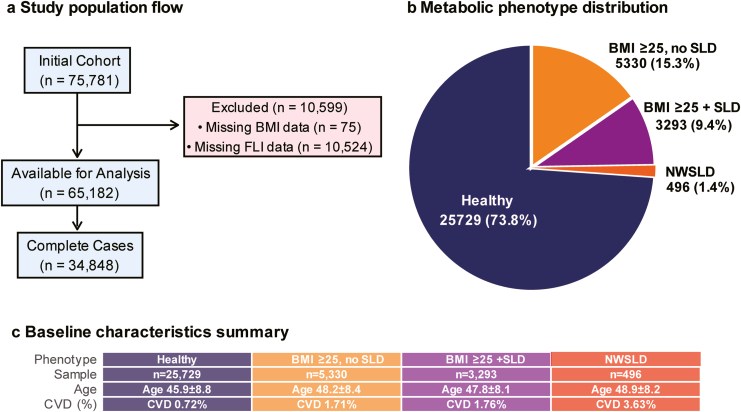
Study flow and metabolic phenotype distribution. (A) Flow diagram showing participant selection from the initial cohort (n = 75,781) to the final analytic sample (n = 34,848 complete cases). (B) Distribution of metabolic phenotypes based on body mass index (BMI) and hepatic steatosis: Healthy (BMI <25 kg/m^2^ and fatty liver index [FLI] <60), BMI ≥25 kg/m^2^ without steatosis, BMI ≥25 kg/m^2^ with steatosis (SLD), and normal-weight individuals with steatotic liver disease (NWSLD). (C) Baseline characteristics of each phenotype. NWSLD accounted for only 1.4% of the population but had the highest cardiovascular disease (CVD) prevalence (3.63%).

From the initial cohort of 75,781 participants, systematic exclusions yielded 34,848 individuals with complete data for analysis (Figure 1A). Among these, 25,729 (73.8%) were classified as healthy; 5330 (15.3%) had elevated BMI without steatotic liver disease, 3293 (9.4%) had elevated BMI with steatotic liver disease, and 496 (1.4%) had NWSLD (Figure 1B, Table 1). The NWSLD group was predominantly male (72.6%) with a mean age of 48.9 ± 8.2 years and a mean BMI of 22.9 ± 1.8 kg/m^2^. Despite normal BMI, individuals with NWSLD exhibited markedly elevated FLI (78.4 ± 18.2), comparable to individuals with elevated BMI and steatotic liver disease (85.7 ± 23.1).

Baseline characteristics of participants included in the complete-case analysis and those excluded due to missing data are presented in Supplementary Table 2. Excluded participants had a higher prevalence of cardiometabolic conditions, including hypertension and dyslipidemia, and lower FLI values compared with included participants.

Despite these differences, the association between NWSLD and prevalent CVD remained materially unchanged in the multiple imputation analysis compared with the complete-case analysis, with similar effect sizes and CIs.

### Baseline Characteristics

Individuals with NWSLD exhibited an intermediate metabolic profile between healthy and elevated BMI phenotypes, characterized by elevated triglycerides (143 ± 89 mg/dL), moderately reduced high-density lipoprotein cholesterol, and elevated liver enzyme levels (Figure 1C, Table 1). Advanced fibrosis (FIB-4 ≥2.67) was rare overall and 0.0% in NWSLD, indicating that fibrosis severity is unlikely to account for the observed NWSLD–CVD association. The prevalence of diabetes in individuals with NWSLD (20.2%) exceeded that of healthy controls (8.4%) but remained lower than in individuals with elevated BMI and steatotic liver disease (28.6%).

### Primary Outcome: Cardiovascular Disease Prevalence (Cross-Sectional Association)

**Figure 2:**
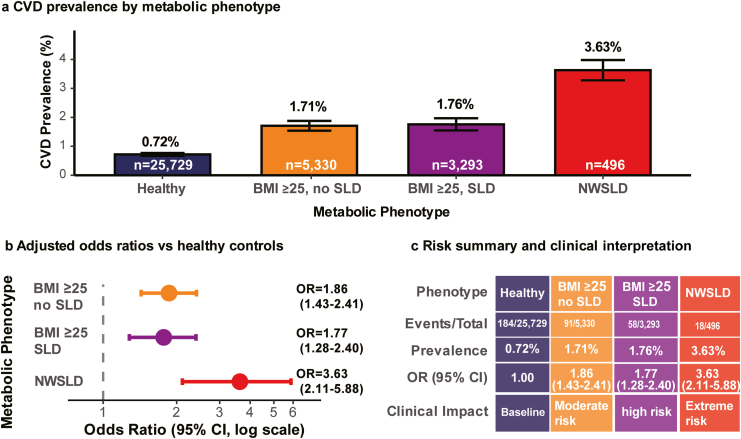
Cardiovascular prevalence by metabolic phenotype. (A) Prevalence of cardiovascular disease (CVD) stratified by metabolic phenotype. (B) Adjusted odds ratios (ORs) for CVD compared with healthy controls (reference). Normal-weight individuals with steatotic liver disease (NWSLD) exhibited the highest OR (3.63; 95% CI: 2.11–5.88). (C) Summary of association metrics and clinical interpretation for each phenotype. NWSLD was associated with the highest prevalence category despite a normal body mass index (BMI).

Individuals with NWSLD demonstrated markedly elevated CVD prevalence (18/496, 3.63%) compared to other metabolic phenotypes (Figure 2A). This risk was 5.0-fold higher than healthy controls (184/25,729, 0.72%) and, paradoxically, 2.1-fold higher than individuals with elevated BMI and steatotic liver disease (58/3,293, 1.76%) (Figure 2B).

After adjustment for age, sex, diabetes, and hypertension (model 2), the NWSLD phenotype was associated with the highest cardiovascular prevalence (odds ratio [OR]: 3.63; 95% CI: 2.11–5.88; *P* < .001) compared to healthy controls. Individuals with elevated BMI without steatotic liver disease (OR: 1.86; 95% CI: 1.43–2.41) and with steatotic liver disease (OR: 1.77; 95% CI: 1.28–2.40) demonstrated similar, more modest risk elevations (Figure 2B and C).

The cardiovascular risk hierarchy defines conventional BMI-based expectations as follows: healthy (0.72%), elevated BMI without steatotic liver disease (1.71%), elevated BMI with steatotic liver disease (1.76%), and NWSLD (3.63%). The absolute risk difference between NWSLD and healthy individuals was 2.91 percentage points (95% CI: 1.85–3.97), corresponding to an NNT of 34 (95% CI: 25–54). This NNT reflects the cross-sectional difference in CVD prevalence and represents identification efficiency rather than an estimate of future event prevention.

Comprehensive multivariable adjustment (model 3) strengthened the association (OR: 4.12; 95% CI: 2.31–6.95; *P* < .001), indicating that the elevated cardiovascular risk in NWSLD was independent of the measured confounders, likely reflecting distinct pathophysiological mechanisms, including concentrated visceral adiposity and hepatic lipotoxicity, despite normal BMI.

### Secondary Outcomes

**Figure 3:**
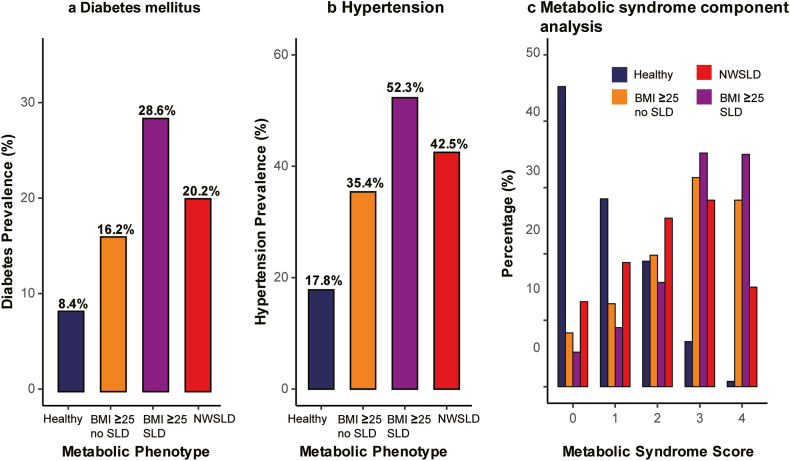
Metabolic abnormalities across phenotypes. (A) Prevalence of diabetes by metabolic phenotype. (B) Prevalence of hypertension. (C) Distribution of metabolic syndrome scores (range: 0–4). Normal-weight individuals with steatotic liver disease (NWSLD) showed disproportionate clustering of cardiometabolic abnormalities despite having a normal body mass index (BMI).

Individuals with NWSLD exhibited elevated diabetes prevalence (20.2%) compared to healthy controls (8.4%, *P* < .001) but lower prevalence than individuals with elevated BMI and steatotic liver disease (28.6%, *P* = .02) (Figure 3A). The prevalence of hypertension in individuals with NWSLD (42.5%) was intermediate, between that of healthy controls (17.8%) and individuals with elevated BMI and steatotic liver disease (52.3%) (Figure 3B).

Metabolic syndrome component analysis revealed distinct risk profiles across phenotypes (Figure 3C). Individuals with NWSLD showed an intermediate prevalence of metabolic syndrome components compared to elevated BMI groups; however, their cardiovascular risk exceeded that of both elevated BMI phenotypes. The CVD-to-diabetes risk ratio differed markedly across phenotypes, with NWSLD showing a disproportionately high cardiovascular risk relative to the diabetes burden (0.18) compared with individuals with elevated BMI and steatotic liver disease (0.06), suggesting distinct pathophysiological mechanisms underlying cardiovascular risk in the NWSLD phenotype.

### Predictive Model Performance

**Figure 4:**
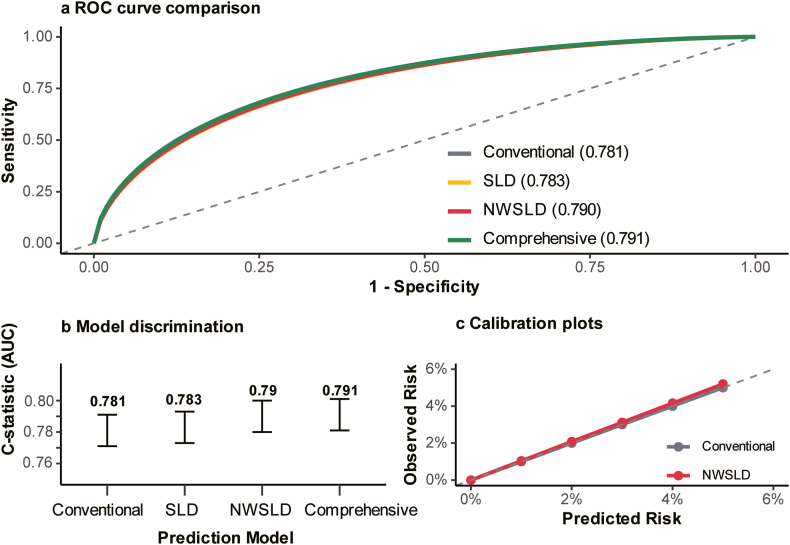
Discriminative performance of models for prevalent CVD. (A) Receiver-operating characteristic (ROC) curves comparing conventional, steatotic liver disease (SLD)-based, normal-weight steatotic liver disease (NWSLD)-inclusive, and comprehensive models. (B) C-statistics (area under the curve [AUC]) for each model; the inclusion of NWSLD significantly improved discrimination (C = 0.790 vs 0.781, *P* < .001). (C) Calibration plots showing agreement between estimated and observed CVD prevalence.

The NWSLD phenotype model demonstrated modestly improved cross-sectional discrimination for prevalent CVD compared with conventional BMI-based approaches (Figure 4A–C, Supplementary Table 3). Receiver-operating characteristic curve analysis revealed incremental improvements across the models, with the C-statistic increasing from 0.781 (95% CI: 0.771–0.791) for the conventional model to 0.790 (95% CI: 0.780–0.800) for the NWSLD model (*P* < .001) (Figure 4A and B). The comprehensive model achieved the highest discrimination (C-statistic: 0.791, 95% CI: 0.781–0.801).

Net reclassification improvement analysis revealed that 4.2% of participants were appropriately reclassified into higher-risk categories using the NWSLD model compared with the conventional BMI-based prediction (*P* = .003). Among individuals who developed CVD, 24.8% were correctly identified as being in the top 5% risk group using the NWSLD model, compared with 20.8% using conventional approaches (*P* =.02). These results indicate that incorporating the NWSLD phenotype improves the model’s ability to discriminate individuals with prevalent CVD who would otherwise be overlooked by BMI-based prediction alone. This supports the exploratory hypothesis that hepatic phenotype information contributes incremental value to cardiometabolic risk assessment beyond anthropometric measures.

All models demonstrated acceptable calibration across risk deciles (Hosmer–Lemeshow *P* > .05), with the NWSLD model showing superior agreement between predicted and observed risks (Figure 4C). Calibration plots confirmed that predicted risks closely matched the observed CVD rates across the full risk spectrum, with accuracy in high-risk individuals.

### Clinical Impact

**Figure 5:**
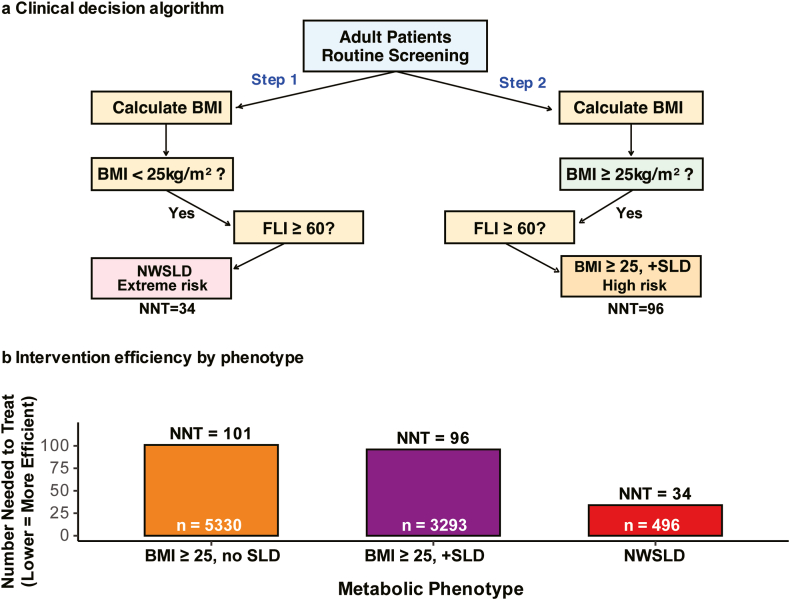
Clinical implementation and efficiency of phenotype-based screening. (A) Proposed clinical decision algorithm integrating the fatty liver index (FLI) and body mass index (BMI) for identifying individuals with a high likelihood of prevalent cardiovascular disease (CVD) among those with normal-weight steatotic liver disease (NWSLD). (B) Number needed to screen (NNS) to prevent one CVD event. NWSLD showed the highest efficiency (NNS = 34) compared with other phenotypes.

The population-attributable risk of NWSLD for CVD was 0.041% (95% CI: 0.025%–0.063%), reflecting both the low prevalence (1.4%) and the extreme risk elevation of the phenotype. Despite the low population prevalence, NWSLD identification enables high-yield interventions, with an NNT of 34 indicating strong clinical benefit, compared to 96 for individuals with elevated BMI and steatotic liver disease and 101 for those with elevated BMI without steatotic liver disease (Figure 5B).

A practical clinical decision algorithm incorporating routine BMI and FLI calculations can systematically identify individuals with NWSLD (Figure 5A). For normal-weight patients, an FLI ≥60 triggers classification as extreme-risk NWSLD, requiring intensive cardiovascular protection equivalent to that provided for established high-risk groups. The algorithm demonstrates high efficiency, as NWSLD identification requires assessing only 1.4% of the population while capturing the highest cardiovascular risk phenotype.

If the 1.4% NWSLD prevalence observed in our cohort is extrapolated to the Japanese adult population (approximately 100 million adults), an estimated 1.4 million individuals would harbor this extreme cardiovascular risk phenotype while appearing metabolically healthy under conventional BMI-based criteria.

### Sensitivity Analyses

Sensitivity analyses confirmed the robustness of the findings across different FLI thresholds, with higher thresholds amplifying the cardiovascular risk. Using FLI ≥50, NWSLD prevalence increased to 2.1%, with a cardiovascular risk of 3.2% (OR: 4.1; 95% CI: 2.8–6.0). Using FLI ≥70, prevalence decreased to 0.8%, with a cardiovascular risk of 4.5% (OR: 5.8; 95% CI: 3.1–10.2). The paradox of elevated cardiovascular risk among individuals with NWSLD persisted across all thresholds.

Alternative BMI cutoff points yielded consistent findings, with the cardiovascular risk hierarchy remaining unchanged across all examined thresholds. Multiple imputation analysis, including participants with missing covariate data (n = 65,182), confirmed the primary findings (OR: 3.41; 95% CI: 2.18–5.12; *P* < .001), demonstrating the robustness of the conclusions to missing data assumptions. Similarly, excluding participants with a prior history of CVD did not materially alter the results (adjusted OR: 2.13; 95% CI: 1.35–3.20; *P* = .00057), indicating that the association between NWSLD and prevalent CVD was not driven by individuals with pre-existing cardiovascular conditions (Supplementary Table 4). After excluding high alcohol intake, the NWSLD–CVD association remained significant and slightly stronger (adjusted OR: 2.51; 95% CI: 1.42–4.13; *P* = .00066), further confirming its robustness. CVD prevalence was 0.385% in high WC patients with NWSLD (n = 260) and 2.758% in those with normal WC (n = 834) (Supplementary Table 5), indicating no monotonic gradient in CVD prevalence by WC.

## Discussion

This analysis of a large population cohort revealed a fundamental paradox: the NWSLD phenotype, affecting 1.4% of individuals, confers a 5-fold elevated cardiovascular prevalence compared to healthy controls and a 2.1-fold higher prevalence than that of traditionally recognized high-risk individuals with elevated BMI and steatotic liver disease.

Our findings challenge the foundational assumption that higher BMI invariably predicts greater cardiovascular risk. The identification of a “hidden” high-risk population among apparently healthy, normal-weight individuals has immediate clinical implications that extend far beyond current risk stratification paradigms. Current guidelines emphasize weight management and intensive monitoring for individuals with elevated BMI, while providing less aggressive risk management for normal-weight patients.^14^ Our data suggest this approach may systematically overlook individuals at the highest risk, potentially contributing to preventable cardiovascular events. The extreme cardiovascular risk in individuals with NWSLD (3.63%) approaches the levels observed in established high-risk populations, such as those with diabetes mellitus or established coronary artery disease,^15^ supporting the need for intensive cardiovascular protection strategies equivalent to secondary prevention protocols. An NNT of 34 for individuals with NWSLD indicates that intensive cardiovascular interventions in this population would be highly cost-effective, comparable to proven strategies, such as statin therapy, in high-risk populations (NNT 30–50).^16^ Healthcare systems should consider developing clinical pathways for NWSLD identification and management through routine FLI calculation for normal-weight patients (Figure 5A). From a healthcare economics perspective, the identification and treatment of NWSLD could prevent approximately 29 cardiovascular events per 1000 patients treated, representing substantial clinical and economic benefits that justify systematic screening implementation.

The NWSLD cardiovascular risk paradox likely reflects the predominance of visceral and subcutaneous adiposity in these individuals, representing a distinct pathophysiological entity.^17^ Unlike generalized weight elevation, visceral adiposity concentrates metabolically active fat around vital organs, promoting insulin resistance, chronic inflammation, and atherogenic dyslipidemia through direct portal circulation effects and enhanced free fatty acid flux.^18^ The hepatic steatosis in NWSLD patients may serve as both a marker and mediator of systemic metabolic dysfunction, creating a vicious cycle of lipotoxicity, oxidative stress, and chronic low-grade inflammation that perpetuates cardiovascular risk. Preserved subcutaneous fat expandability in individuals with NWSLD may paradoxically worsen metabolic outcomes by limiting healthy fat storage capacity and forcing lipid overflow into ectopic sites—such as the liver, muscle, pancreas, and pericardium—resembling a lipodystrophy-like phenotype characterized by impaired subcutaneous fat storage and ectopic fat deposition.^18^ This mechanism explains the severe metabolic consequences despite normal total body weight and BMI, as these individuals essentially experience “metabolic crowding” at lower total adiposity levels. The ectopic fat accumulation in vital organs disrupts normal cellular function and promotes local inflammation, contributing to organ-specific insulin resistance and metabolic dysfunction. Additionally, individuals with NWSLD may have genetic predispositions to inefficient subcutaneous fat storage, enhanced visceral fat accumulation, and altered adipokine secretion patterns, leading to metabolic dysfunction at low total adiposity levels.^19^ WC stratification within NWSLD did not display a monotonic increase in CVD prevalence. This pattern may reflect heterogeneity in metabolic health among lean individuals, reinforcing that hepatic steatosis can capture risk not fully represented by BMI or WC alone. Polymorphisms in genes regulating lipid metabolism and adipose tissue function may contribute to this phenotype by affecting the body’s ability to safely store excess energy. The high male predominance (72.6%) in our NWSLD cohort supports this mechanism, as males typically demonstrate preferential visceral fat deposition and reduced subcutaneous fat storage capacity compared to females, making them more susceptible to metabolic dysfunction at lower BMI levels. Minimal advanced fibrosis in NWSLD (FIB-4 ≥2.67: 0.0%) suggests that metabolic–vascular mechanisms, rather than fibrotic burden, underlie the excess CVD prevalence.

The paradoxical finding that individuals with NWSLD have a higher cardiovascular risk than those with elevated BMI and steatotic liver disease represents a novel discovery not previously reported at the population scale. Recent meta-analyses of metabolically unhealthy normal-weight individuals reported a 2–3-fold increase in cardiovascular risk^20^; however, these studies used varied definitions of metabolic dysfunction and had smaller sample sizes. Asian population studies have consistently identified lower BMI thresholds for the development of metabolic dysfunction, supporting our findings of extreme cardiovascular risk in normal-weight individuals. However, most previous Asian studies have examined diabetes or metabolic syndrome rather than CVD endpoints, highlighting the novelty of our cardiovascular-focused analysis. Our cross-sectional prevalence findings complement longitudinal evidence reporting lower CVD event incidence but higher CVD mortality in lean metabolic dysfunction-associated steatotic liver disease^21^; differences in study design (prevalence vs incidence) and endpoints (nonfatal vs fatal) likely explain the divergence while collectively underscoring the distinct vulnerability of lean steatotic liver disease.

Our findings have significant implications for the development of global strategies to prevent CVD, particularly in regions experiencing increasing rates of metabolic dysfunction despite stable or declining BMI trends. The current WHO and national guidelines emphasize BMI-based risk stratification, potentially missing high-risk populations in countries with a lower average BMI but high rates of metabolic dysfunction.^22^ The NWSLD phenotype may be particularly prevalent in Asian populations because of their genetic susceptibility to visceral adiposity at lower BMI. Still, similar patterns may emerge globally as metabolic dysfunction increases independently of weight gain. Public health screening programs should incorporate metabolic phenotyping beyond BMI assessment, using accessible tools that can identify high-risk individuals regardless of their anthropometric profile. The FLI requires only routine laboratory parameters and anthropometric measurements, making its implementation feasible in resource-limited settings without requiring advanced imaging or specialized equipment. Implementation feasibility studies in diverse healthcare systems could inform optimal screening strategies and identify barriers to adoption. Cost-effectiveness analyses of NWSLD screening strategies, considering both direct medical costs and productivity losses from cardiovascular events, would support evidence-based policy decisions and justify resource allocation for systematic screening programs. Furthermore, our findings suggest that prevention campaigns focusing solely on BMI reduction may not adequately address cardiovascular risk in large populations, particularly in regions where normal-weight metabolic dysfunction is increasingly common. Interventions targeting visceral adiposity reduction through specific dietary patterns, structured exercise programs emphasizing resistance training, and lifestyle modifications may be more effective than traditional weight-loss interventions in NWSLD populations, requiring a fundamental shift in public health messaging and intervention strategies.

Our findings highlight the potential of phenotype-driven precision medicine, in which markers of metabolic dysfunction replace crude anthropometric measures to refine cardiovascular risk assessment. The NWSLD phenotype exemplifies this paradigm shift toward precision medicine approaches for cardiovascular risk assessment. Machine learning findings are exploratory and illustrate that incorporating hepatic phenotype (eg, NWSLD) may capture metabolic profiles not represented by BMI and modestly improve cross-sectional discrimination for prevalent CVD; prospective studies are warranted before any clinical implementation. Future studies should investigate integrating additional biomarkers, genetic factors, and imaging parameters to refine NWSLD identification and risk stratification further.

Digital health platforms can facilitate NWSLD identification through automated FLI calculations and risk assessments, enabling population-scale screening and early intervention. Routine FLI screening could enable early identification of NWSLD, preventing oversight of cardiovascular risk among normal-weight individuals.

This study has some limitations. The cross-sectional design precludes causal inference and temporal sequencing, limiting our ability to determine whether NWSLD precedes CVD development or represents a consequence of subclinical CVD. Accordingly, the present results should be interpreted as associations with prevalent CVD rather than prospective risk estimates and do not imply causality or future risk prediction. Longitudinal studies with repeated phenotype assessments are needed to establish causality and quantify absolute risk over time, including the natural history of NWSLD progression and potential reversibility with targeted interventions. Although reverse causality cannot be entirely excluded, the association between NWSLD and prevalent CVD remained significant after excluding participants with prior CVD, suggesting that the observed relationship is unlikely to be solely explained by post-CVD metabolic changes or weight loss. This persistence after removing heavy drinkers indicates that the association is independent of alcohol-related hepatic injury. CVD ascertainment relied on clinical diagnosis and medical history rather than standardized adjudication using contemporary imaging modalities or biomarkers, potentially leading to outcome misclassification. However, any misclassification is likely nondifferential across phenotype groups, potentially underestimating the true effect sizes. Future studies should incorporate standardized cardiovascular imaging and cardiac biomarkers to enhance outcome precision. Hepatic steatosis was assessed using a validated index rather than direct imaging, although the FLI demonstrated strong concordance with ultrasonography and biopsy findings. Although more precise quantitative methods, such as magnetic resonance imaging proton density fat fraction, are available for steatosis grading,^13^ the FLI remains the most feasible approach for large-scale epidemiological studies. Because FLI incorporates BMI and WC, potential circularity may be considered when stratifying participants by BMI categories. However, FLI also integrates biochemical markers of metabolic dysfunction, thus reflecting hepatic and metabolic characteristics beyond anthropometric measures alone. Importantly, our sensitivity analyses using alternative FLI thresholds yielded consistent results, supporting the robustness of the phenotype classification and mitigating concerns that the observed associations were driven by BMI-related circularity. Nevertheless, residual conceptual overlap between anthropometric and metabolic components cannot be entirely excluded; future studies using imaging-based steatosis quantification may further strengthen phenotype precision. The health screening population, while large and well-characterized, may not be fully representative of the general population due to selection bias toward health-conscious individuals, limiting generalizability to broader populations with different healthcare access patterns. Moreover, baseline differences were observed between participants included in the complete-case analysis and those excluded due to missing data (Supplementary Table 2), indicating that missingness may not have been completely at random. Although this raises the possibility of selection bias, the concordant findings obtained from the multiple imputation analysis mitigate concerns regarding substantial bias affecting the primary conclusions. Furthermore, because NNT was derived from cross-sectional differences in CVD prevalence rather than incident outcomes, it should be interpreted as a measure of identification efficiency rather than prevention of future cardiovascular events. Prospective longitudinal studies are required to validate its prognostic utility for incident CVD. Unmeasured confounding factors remain possible, although our comprehensive adjustment for lifestyle factors, laboratory parameters, and comorbidities makes it unlikely that residual confounding explains the large effect sizes observed. Prospective longitudinal studies should examine the incidence of cardiovascular events rather than their prevalence to establish temporal relationships and quantify absolute risk.

## Conclusion

Individuals with NWSLD represent a previously unrecognized population at extreme cardiovascular risk, challenging fundamental assumptions underlying BMI-based risk assessments. This phenotype is a distinct high-risk category, warranting intensive cardiovascular protection strategies equivalent to those used for established high-risk populations. Clinical practice must evolve to integrate metabolic phenotyping beyond BMI-based assessment to optimize cardiovascular risk stratification and prevention. Consistent with recent work highlighting the limitations of BMI-centric cardiovascular algorithms in metabolic dysfunction-associated steatotic liver disease,^23^ our data suggest that integrating hepatic phenotype information (eg, NWSLD) could improve cross-sectional identification of high-burden individuals beyond BMI alone. This population-scale validation of the NWSLD concept supports a paradigm shift toward precision medicine approaches that prioritize metabolic dysfunction over anthropometric criteria in cardiovascular risk assessment. The extreme cardiovascular risk in individuals with NWSLD demands immediate clinical recognition and intensive protective strategies to prevent potentially avoidable cardiovascular events in this vulnerable population.

## Supplementary Materials

Supplementary Tables 1–3

Extended PDF

## References

[1] Ndumele C.E., Rangaswami J., Chow S.L. (2023). Cardiovascular-kidney-metabolic health: a presidential advisory from the American Heart Association. Circulation.

[2] Sebastian S.A., Padda I., Johal G. (2024). Cardiovascular-Kidney-Metabolic (CKM) syndrome: a state-of-the-art review. Curr Probl Cardiol.

[3] Correa-Rodriguez M., Gonzalez-Ruiz K., Rincon-Pabon D. (2020). Normal-weight obesity is associated with increased cardiometabolic risk in young adults. Nutrients.

[4] Schulze M.B., Stefan N. (2024). Metabolically healthy obesity: from epidemiology and mechanisms to clinical implications. Nat Rev Endocrinol.

[5] Powell-Wiley T.M., Poirier P., Burke L.E. (2021). Obesity and cardiovascular disease: a scientific statement from the American Heart Association. Circulation.

[6] Meyersohn N.M., Mayrhofer T., Corey K.E. (2021). Association of hepatic steatosis with major adverse cardiovascular events, independent of coronary artery disease. Clin Gastroenterol Hepatol.

[7] Li Z., Daniel S., Fujioka K. (2023). Obesity among Asian American people in the United States: a review. Obesity (Silver Spring).

[8] Wu Y., Li D., Vermund S.H. (2024). Advantages and limitations of the body mass index (BMI) to assess adult obesity. Int J Environ Res Public Health.

[9] Mehta S., Zhao J., Poppe K. (2022). Cardiovascular preventive pharmacotherapy stratified by predicted cardiovascular risk: a national data linkage study. Eur J Prev Cardiol.

[10] Bedogni G., Bellentani S., Miglioli L. (2006). The Fatty Liver Index: a simple and accurate predictor of hepatic steatosis in the general population. BMC Gastroenterol.

[11] Koehler E.M., Schouten J.N., Hansen B.E. (2013). External validation of the fatty liver index for identifying nonalcoholic fatty liver disease in a population-based study. Clin Gastroenterol Hepatol.

[12] Huang X., Xu M., Chen Y. (2015). Validation of the fatty liver index for nonalcoholic Fatty liver disease in middle-aged and elderly Chinese. Medicine (Baltimore).

[13] Hirooka M., Ogawa S., Koizumi Y. (2024). iATT liver fat quantification for steatosis grading by referring to MRI proton density fat fraction: a multicenter study. J Gastroenterol.

[14] American Diabetes Association Professional Practice Committee (2024). 8. Obesity and weight management for the prevention and treatment of type 2 diabetes: standards of care in diabetes-2024. Diabetes Care.

[15] Siam N.H., Snigdha N.N., Tabasumma N. (2024). Diabetes mellitus and cardiovascular disease: exploring epidemiology, pathophysiology, and treatment strategies. Rev Cardiovasc Med.

[16] Chou R., Cantor A., Dana T. (2022). Statin use for the primary prevention of cardiovascular disease in adults: updated evidence report and systematic review for the US preventive services task force. JAMA.

[17] Tang A., Ng C.H., Phang P.H. (2023). Comparative burden of metabolic dysfunction in lean NAFLD vs non-lean NAFLD - a systematic review and meta-analysis. Clin Gastroenterol Hepatol.

[18] Kabir M., Catalano K.J., Ananthnarayan S. (2005). Molecular evidence supporting the portal theory: a causative link between visceral adiposity and hepatic insulin resistance. Am J Physiol Endocrinol Metab.

[19] Feldman A., Eder S.K., Felder T.K. (2017). Clinical and metabolic characterization of lean casian subjects with non-alcoholic fatty liver. Am J Gastroenterol.

[20] Putra I.C.S., Kamarullah W., Prameswari H.S. (2022). Metabolically unhealthy phenotype in normal weight population and risk of mortality and major adverse cardiac events: a meta-analysis of 41 prospective cohort studies. Diabetes Metab Syndr.

[21] Huo Z., Chen Y., Huang Y. (2026). Long-term prognosis of lean MASLD: evidence from three population-based prospective cohorts. Gut.

[22] WHO Expert Consultation (2004). Appropriate body-mass index for Asian populations and its implications for policy and intervention strategies. Lancet.

[23] Barritt ASt, Tapper E.B., Newsome P.N. (2026). Cardiovascular risk assessment tools are insufficient for patients with metabolic dysfunction associated steatotic liver disease. Am J Gastroenterol.

