# Supplementary material for: The Cardiovascular Risk Paradox in Normal-Weight Steatotic Liver Disease Beyond Body Mass Index–Based Detection
Source: Gastro Hep Adv. 2026 May 5;5(7):100988. doi: 10.1016/j.gastha.2026.100988 (PMC13234475; doi:10.1016/j.gastha.2026.100988)
Supplement: Supplementary Tables 1–3 [file mmc1.pdf]

Supplementary Table 1. Baseline characteristics of participants included in the complete-case analysis and those excluded due to missing data

Variable	Excluded (n = 10,599)	Included (n = 65,182)	Standardized Mean Difference
Age, years	48.61 ± 9.69	47.24 ± 9.25	0.14
Male, n (%)	5,130 (48.4%)	33,373 (51.2%)	0.06
BMI, kg/m²	22.68 ± 3.66	22.94 ± 3.60	0.07
FLI	14.21 ± 16.63	24.39 ± 24.43	0.49
Hypertension, n (%)	4,706 (44.4%)	16,817 (25.8%)	0.39
Diabetes mellitus, n (%)	784 (7.4%)	8,930 (13.7%)	0.21
Dyslipidemia, n (%)	7,642 (72.1%)	28,745 (44.1%)	0.57
Cardiovascular disease, n (%)	95 (0.9%)	652 (1.0%)	0.01

Data are presented as mean ± standard deviation for continuous variables and number (percentage) for categorical variables.

Standardized mean differences (SMDs) are presented as absolute values; values ≥0.20 indicate meaningful imbalance between groups.

Supplementary Table 2. Operational definition and questionnaire items used for cardiovascular disease (CVD) classification

Disease category	Ascertainment method	Questionnaire item (English translation)
Coronary artery disease	Institutional notification (primary source)	Notification from treating institution to the health screening center confirming coronary angiography and/or coronary revascularization
Coronary artery disease	Self-report (if no institutional notification)	Have you ever undergone coronary angiography or coronary revascularization?
Cerebrovascular disease	Institutional notification (primary source)	Notification from treating institution to the health screening center confirming imaging-based diagnosis and treatment of cerebrovascular disease
Cerebrovascular disease	Self-report (if no institutional notification)	Have you ever been diagnosed with ischemic or hemorrhagic stroke based on imaging?

Items were originally administered in Japanese as part of the standardized health screening program. English translations are provided for reference.

Supplementary Table 3. Cardiovascular risk and model performance

(A) Cardiovascular Risk by Phenotype

**Phenotype**	**CVD Events / Total**	**Prevalence (%)**	**Unadjusted OR (95% CI)**	**Adjusted OR (95% CI)**	***P-value***
Healthy	184 / 25,729	0.72	1.00 (reference)	1.00 (reference)	---
Elevated BMI, no SLD	91 / 5,330	1.71	2.40 (1.86–3.09)	1.86 (1.43–2.41)	*<0.001*
Elevated BMI + SLD	58 / 3,293	1.76	2.47 (1.83–3.33)	1.77 (1.28–2.40)	*<0.001*
NWSLD	18 / 496	3.63	5.17 (3.12–8.36)	3.63 (2.11–5.88)	*<0.001*

CVD: cardiovascular disease; BMI: body mass index; SLD: steatotic liver disease; NWSLD: normal-weight steatotic liver disease; OR: odds ratio; CI: confidence interval.

Adjusted OR: Model 2 adjustment for age, sex, diabetes, and hypertension.

(B) Cardiovascular Risk Prediction Model Performance

**Model**	**C-statistic (95% CI)**	**AIC**	**NRI (95% CI)**	***P-value***
BMI-based (conventional)	0.781 (0.771–0.791)	3573.4	Reference	---
SLD-enhanced	0.783 (0.773–0.793)	3569.4	0.018 (0.003–0.041)	*0.020*
NWSLD phenotype	0.790 (0.780–0.800)	3545.7	0.042 (0.015–0.075)	*0.003*
Comprehensive model	0.791 (0.781–0.801)	3540.1	0.045 (0.017–0.081)	*0.002*

C-statistic: area under the receiver operating characteristic curve; AIC: Akaike Information Criterion; NRI: net reclassification improvement.

Supplementary Table 4. Sensitivity analysis excluding participants with prior cardiovascular disease.

Variable	Adjusted OR	95% CI	*P-value*
Elevated BMI, no SLD	1.96	1.54–2.47	*$2.4×10^{-8}$*
Elevated BMI + SLD	1.74	1.30–2.30	*$1.5×10^{-4}$*
NWSLD	2.51	1.42–4.13	*$6.6×10^{-4}$*
Age (per year)	1.10	1.09–1.11	*<0.001*
Male sex	1.26	1.02–1.57	*0.033*
Diabetes mellitus	1.27	0.99–1.60	*0.053*
Hypertension	1.97	1.59–2.45	*<0.001*

NWSLD, normal-weight steatotic liver disease; SLD, steatotic liver disease; BMI, body mass index; FLI, fatty liver index; OR, odds ratio; CI, confidence interval; DM, diabetes mellitus; HTN, hypertension.

Elevated BMI: body mass index ≥25 kg/m² (Asia-Pacific criteria).

SLD (steatotic liver disease): fatty liver index (FLI) ≥60.

NWSLD (normal-weight steatotic liver disease): BMI <25 kg/m² and FLI ≥60.

Healthy reference: BMI <25 kg/m² and FLI <60 (no steatosis).

Supplementary Table 5. Cardiovascular disease prevalence by waist circumference within the NWSLD group.

Waist circumference category	n	CVD cases	Prevalence (%)
High WC (≥90 cm men, ≥80 cm women)	260	1	0.385
Normal WC (<90 cm men, <80 cm women)	834	23	2.758

CVD, cardiovascular disease; WC, waist circumference; NWSLD, normal-weight steatotic liver disease; FLI, fatty liver index; BMI, body mass index.

NWSLD (normal-weight steatotic liver disease): BMI <25 kg/m² and FLI ≥60.

High waist circumference (WC): ≥90 cm in men or ≥80 cm in women (WHO Asia–Pacific criteria).

Normal WC: below these thresholds.
